# Expression of GABAergic Receptors in Mouse Taste Receptor Cells

**DOI:** 10.1371/journal.pone.0013639

**Published:** 2010-10-26

**Authors:** Margaret R. Starostik, Michelle R. Rebello, Kellie A. Cotter, Akos Kulik, Kathryn F. Medler

**Affiliations:** 1 Department of Biological Sciences, University at Buffalo, The State University of New York, Buffalo, New York, United States of America; 2 Institute of Anatomy and Cell Biology, Department of Neuroanatomy, University of Freiburg, Freiburg, Germany; Duke University, United States of America

## Abstract

**Background:**

Multiple excitatory neurotransmitters have been identified in the mammalian taste transduction, with few studies focused on inhibitory neurotransmitters. Since the synthetic enzyme glutamate decarboxylase (GAD) for gamma-aminobutyric acid (GABA) is expressed in a subset of mouse taste cells, we hypothesized that other components of the GABA signaling pathway are likely expressed in this system. GABA signaling is initiated by the activation of either ionotropic receptors (GABA_A_ and GABA_C_) or metabotropic receptors (GABA_B_) while it is terminated by the re-uptake of GABA through transporters (GATs).

**Methodology/Principal Findings:**

Using reverse transcriptase-PCR (RT-PCR) analysis, we investigated the expression of different GABA signaling molecules in the mouse taste system. Taste receptor cells (TRCs) in the circumvallate papillae express multiple subunits of the GABA_A_ and GABA_B_ receptors as well as multiple GATs. Immunocytochemical analyses examined the distribution of the GABA machinery in the circumvallate papillae. Both GABA_A_-and GABA_B_- immunoreactivity were detected in the peripheral taste receptor cells. We also used transgenic mice that express green fluorescent protein (GFP) in either the Type II taste cells, which can respond to bitter, sweet or umami taste stimuli, or in the Type III GAD67 expressing taste cells. Thus, we were able to identify that GABAergic receptors are expressed in some Type II and Type III taste cells. Mouse GAT4 labeling was concentrated in the cells surrounding the taste buds with a few positively labeled TRCs at the margins of the taste buds.

**Conclusions/Significance:**

The presence of GABAergic receptors localized on Type II and Type III taste cells suggests that GABA is likely modulating evoked taste responses in the mouse taste bud.

## Introduction

Chemosensory reception in the peripheral sensory organs of taste is influenced by neuroactive molecules that ultimately regulate signaling to and from taste buds. Taste receptor cells (TRCs), housed in taste buds, transmit signals by forming synaptic connections with sensory afferent fibers and perhaps even with other TRCs within the taste bud [1], [2], [3]. To date, serotonin (5-hydroxytryptamine; 5HT) and ATP [4], [5] have been most definitively identified within the taste bud as neurotransmitters through anatomical localizations, physiological observations, and pharmacological data. Histochemical and immunocytochemical techniques have shown that 5HT is expressed in a subset of Type III TRCs from circumvallate and foliate papillae in mammals as well as in amphibian taste buds [6], [7], [8], [9], [10], [11]. Other evidence currently exists for acetylcholine, adrenergic neurotransmission, neuropeptides, glutamate, and γ-aminobutyric acid (GABA) expression in taste buds [12], [13], [14], [15], [16], [17], [18], [19], [20], [21], [22]. However, the physiological roles for most of these neurotransmitters have not been well defined. Recently, it was determined that expression of glutamate decarboxylase (GAD67), an enzyme which converts glutamate into GABA [23], is expressed in a subset of Type III taste cells in mice [24], [25]. While these findings revealed a useful marker to enable the identification of taste cells with chemical synapses, it also indicated that GABA is likely produced and released by these cells.

GABA is well known as an inhibitory mediator of neural transmission in the mammalian central nervous system [26], [27], [28]. GABA acts through two distinct types of receptors: ionotropic and metabotropic [29]. Ligand-gated GABA_A_ ion channels are pentameric channels comprised of a combination of subunit subtypes (α_1–6_, β_1–3_, γ_1–3_, δ, ε, π, θ), which determine specific pharmacological and gating properties [30], [31], [32]. Activation of these channels generates the fast inhibitory actions of GABA [33], [34]; the slower, more modulatory actions of GABA are mediated by heterodimers of GABA_B_ receptors which are G-protein coupled receptors [35], [36]. GABAergic transmission is terminated by the uptake of GABA through GABA transporters (GATs). Molecular cloning studies have revealed the existence of four subtypes of GATs, GAT1-4, which are uniquely distributed in different cell types and regions [37], [38], [39], [40].

In *Necturus maculosus*, immunocytochemistry revealed GABA staining in nerve fibers innervating taste buds and in varicose axons penetrating the surrounding non-taste epithelium [41]. However, further studies were unable to evoke GABA release in response to stimulus and studies to ascertain a role for GABA neurotransmission in mudpuppy taste cells were inconclusive [16], [42]. Immunocytochemical analysis of rat taste buds determined that GABA immunoreactive taste cells are present throughout the taste buds and that GAT3 is the primary transporter responsible for GABA uptake in these cells. This study, however, did not attempt to identify any GABAergic receptors that may be expressed in these taste buds [43]. Recently, Cao et al. [12] reported on the expression of GABA_A_α1 and GABA_B_ receptors in rat taste buds and provided evidence that GABA has a physiological role in rat taste cells [12]. Based on these studies and the recent reports of GAD expression in mouse taste cells [24], [25], we reasoned that GABA likely contributes to the transmission of taste-induced signals in mouse taste buds. Using RT-PCR and immunocytochemical analysis, we investigated the expression and localization of GABA receptors and transporters in mouse taste cells to determine if the appropriate molecular machinery is in place to enable GABA to act as a neurotransmitter in this system.

## Materials and Methods

### Animals

Adult FVB IP_3_R3-tauGFP mice were used in the experiments. These transgenic animals express the green fluorescent protein (GFP) in taste cells with the IP_3_R3 protein and can be used to identify Type II taste cells [44]. We also used a transgenic mouse line that expresses GFP in GAD67 expressing taste cells which identify a subset of Type III taste cells [24], [25]. These mice have a CB6F1/J background and were purchased from Jackson Labs (cat#007677). Both sexes of mice were used and animals ranged in age from 6 weeks to 6 months. All studies were approved and animals were cared for in compliance with the University at Buffalo Animal Care and Use Committee under protocol number #BIO010174N.

### Taste receptor cell collection

Mice were euthanized with carbon dioxide and cervical dislocation. Tongues were removed from animals followed by injection under the lingual epithelium with 100 µl of an enzymatic solution containing 0.6 mg of collagenase B (Roche, Indianapolis, IN), 3 mg of dispase II (Roche), and 1 mg of trypsin inhibitor (Sigma, St. Louis, MO) per milliliter of Tyrode's solution (140 mM NaCl, 5 mM KCl, 1 mM MgCl_2_, 3 mM CaCl_2_, 10 mM HEPES, 10 mM glucose, and 1 mM pyruvic acid. Adjusted to pH 7.4 with 1 mM NaOH or 1 mM HCl). Tongues were incubated in oxygenated Tyrode's solution for 20 min before the epithelium was peeled from the connective and muscular tissue. The peeled epithelium was incubated for 30 min in Ca^2+^-free Tyrode's solution (140 mM NaCl, 5 mM KCl, 10 mM HEPES, 2 mM BAPTA, 10 mM glucose and 1 mM pyruvic acid. Adjusted to pH 7.4 with 1 mM NaOH or 1 mM HCl) before taste buds were removed with a capillary pipette using gentle suction and frozen for later analysis.

### RNA isolation and sample analysis

Total RNA was extracted from multiple isolated taste buds from the circumvallate papillae, non-gustatory lingual epithelium, and the brain (cerebellum) using Trizol (Invitrogen Corporation, Carlsbad, CA) according to the manufacturer's protocol. Taste buds and epithelia tissue were each collected from two mice and combined for RNA isolation. Unamplified total RNA was DNAse treated and then subjected to reverse transcription using Superscript III (Invitrogen) to yield cDNA. We tested the quality of the cDNA using a PCR for glyceraldehyde 3-phosphate dehydrogenase (GAPDH), a housekeeping gene that has constitutive and wide-spread expression [45], [46]. Only samples that correctly amplified GAPDH products and lacked genomic contamination were used for subsequent experiments (see Figure S1). All materials were purchased from Fermentas (Glen Burnie, MD) unless otherwise noted.

### PCR Analysis

Primers were custom made (Integrated DNA Technologies Inc., Coralville, IA) for the GABA_A_α subunits using the primer design tool OLIGO (Molecular Biology Insights Inc., Cascade, CO) with sequence files deposited in the Entrez Nucleotides database (www.ncbi.nlm.nih.gov). Primers for GABA transporters (GAT), the subunits of GABA_A_ β and GABA_B_ receptors were taken from previously published studies [47], [48], [49], [50]. PCRs were performed in 25 µL reactions with 2 µL cDNA. Samples were run for 40 cycles at 95°C for 30 sec, specific annealing temperatures for 45 sec (see Table 1) and 72°C for 90 sec. PCR products were separated by electrophoresis on a 1% agarose gel. All PCR products were gel purified and subjected to DNA sequencing to confirm identity. Experiments were repeated three to five times with different cDNA samples to confirm the findings.

**Table 1:** Primer sequences for mouse GABA_A_ and GABA_B_ subunits and GABA transporters.

Primers	GenBank Access Number	Sequence of Primer	Amplicon size (bp)	Annealing (°C)
GABA_A_ α_1_	NM_010250.4	F 5′-cggctaaacaaccttatgg-3′	455	60.0
		R 5′-attatgcacggcagatatgt-3′		
GABA_A_ α_2_	NM_008066.3	F 5′-cagtccaagccgaatgt-3′	498	60.0
		R 5′-cagagaacacaaacgcataa-3′		
GABA_A_ α_3_	NM_008067.3	F 5′-cacgcctgaatcagtatga-3′	511	46.1
		R 5′-ttggccagattgataggata-3′		
GABA_A_ α_4_	NM_010251.2	F 5′-aatacagatgccgaccag-3′	699	60.0
		R 5′-gaatcttgcgaggacattag-3′		
GABA_A_ α_5_	NM_176942.4	F 5′-aatagagagcccgtgataaa-3′	465	60.0
		R 5′-tcattaacagcgtgtaccc-3′		
GABA_A_ α_6_	NM_001099641.1 (variant 1)	F 5′-gcaaagccctcagtagaac-3′	358	60.0
	NM_008068.2 (variant 2)	R 5′-gcccatacatacctattcca-3′		
GABA_A_ β_1_	NM_008069.4	F 5′-acagtacaaaatcgagagagtttg-3′	665	61.4
		R 5′-tccaccttcttggacaccatcttg-3′		
GABA_A_ β_2_	NM_008070.3	F 5′-ataaactcatcaccaagaaagttg-3′	514	58.9
		R 5′-aagtcccattactgcttctgatgt-3′		
GABA_A_ β_3_	NM_008071.3 (variant 1)	F 5′-gagcaccgtctggtctccagga-3′	415	55.8
	NM_001038701.1 (variant 2)	R 5′-cgatcattcttggccttggctgt-3′		
GABA_B1_	NM_019439.3	F 5′-ctgcccggatgtggaacctta-3′	427	63.1
		R 5′-tcagcataccacccgatgaga-3′		
GABA_B2_	NM_001081141.1	F 5′-atcgagcagatccgcaacgag-3′	993	63.1
		R 5′-acacaacttgacccgtgaccc-3′		
GAT1	NM_178703.3	F 5′-gaagccagcggagacagtttctg-3′	697	62.1
		R 5′-gagcagcaagaaggagacctcct-3′		
GAT2	NM_133661.3	F 5′-tcctctccagccaaacaagaact-3′	438	63.3
		R 5′-atgcaggcttgttagctgctgca-3′		
GAT3	NM_144512.2	F 5′-aactgctcctgcgacaccgatga-3′	354	61.0
		R 5′-ttggatttaatgacatggaaggaag-3′		
GAT4	NM_172890.3	F 5′-ggagttcgtgttgagcgtag-3′	681	65.0
		R 5′-gaacttgatgccttcagaggc-3′		

### Immunocytochemistry

Mice were deeply anesthetized and then perfused transcardially with a solution of 0.025% heparin and 1% sodium nitrite followed by 4% buffered paraformaldehyde/0.1 M phosphate buffer (PB), pH 7.2. Tongues were removed and post-fixed for 1 hour in 4% paraformaldehyde/0.1 M PB, then transferred in 20% sucrose solution at 4°C overnight for cryoprotection. Tongues that were used for the GABA_B_ antibody experiments were immersion fixed overnight in 4% buffered paraformaldehyde/0.1 M phosphate buffer (PB), pH 7.2 at 4°C. 40 µm sections of mouse circumvallate papillae were cut, washed in 0.1 M phosphate buffered saline (PBS, pH 7.2) and then blocked for 1 hour at RT.

Primary antibodies used in this study were: rabbit polyclonal anti-GABA_A_ Rα1 (1∶100, Millipore, Temecula, CA) [12], rabbit polyclonal anti-GABA_B_ R1 subtype (1∶200) [51], [52], [53], rabbit polyclonal anti-GABA_B_ R2 subtype (1∶200) [51], [52], and rabbit polyclonal anti-GABA transporter-3 (1∶100, Millipore) [54], [55], [56]. Sections were incubated overnight in primary antibodies at 4°C, washed with PBS and then incubated for 2 hours at RT in the dark with the secondary cy-5 anti-rabbit antibody (1∶250; Jackson ImmunoResearch Laboratories Inc., West Grove, PA). Following this incubation, sections were washed and mounted on slides using Flouromount G (Southern Biotechnology Associates, Birmingham, AL) and coverslipped. Negative controls lacking primary antibody were run with each experiment. A blocking peptide for anti-GABA_A_ Rα1 (corresponding to amino acids 28–43) was pre-incubated with the antibody (1 µg peptide with 1 µg antibody) and staining was eliminated.

Sections were viewed with a three-channel laser scanning confocal with Krypton-Argon lasers on a Nikon Diaphot 200. Images were sequentially captured with a cooled CCD camera, and Axiovision software was used for data acquisition. Images were processed using Adobe Photoshop CS software adjusting only brightness and contrast. Settings for the negative control sections were matched to the immunoreactive sections, both for the initial collection of the images and during the final adjustment for brightness and contrast.

### Antibody characterization

#### Anti-GABA_A_α1

A rabbit polyclonal anti-GABA_A_ Rα1 (Millipore, cat #AB5592) was produced against the peptide corresponding to amino acids 28–43 from mouse or rat GABA(A) α1 subunit (Accession P18504). This antibody recognizes a single band of molecular size 51kD in western blots of brain and taste tissue [12]. In addition, pre-absorption with the control antigen eliminates staining.

#### Anti-GABA_B1_

A rabbit polyclonal anti-GABA_ B1_ antibody was produced against amino acid residues 901–960 of rat GABA_ B1_ which recognizes both GABA_ B1_R1a and GABA_ B1_ R1b isoforms in the brain. In COS expressing cells, this antibody reacted specifically with GABA_ B1_ and did not cross react with GABA_B2_. Labeling was blocked when the antibody was pre-absorbed with the antigen [53]. Control electron microscopy experiments using a GABA_ B1_ knock out mouse revealed no labeling.

#### Anti-GABA_B2_

A rabbit polyclonal anti-GABA_B2_ antibody was produced against amino acid residues 844–892 of GABA_B2_ which labeled a single band of 110 kDa in the brain. In receptor expressing COS cells, this antibody reacted specifically with GABA_B2_ and did not cross react with GABA_B1a_ or GABA_B1b_ [52]. Control electron microscopy experiments using a GABA_B2_ knock out mouse brain revealed no labeling. Additional electron microscopy comparing the labeling patterns of this antibody to another GABA_B2_ antibody that was raised against a different epitope [53] revealed similar sub-cellular distribution within hippocampal neurons for both antibodies.

#### Anti-GAT3

A rabbit polyclonal anti-rat GAT3 (Millipore cat #AB1574) was raised against peptide corresponding to amino acids 607–627 in the C terminus of rat GAT3. Anti-GAT3 recognizes a single band at 71kD in the mouse brain and retina and staining was eliminated after preadsorption with the cognate-peptide [54], [55], [56].

## Results

Results from the RT-PCR analysis of the GABA receptors and transporters revealed that multiple isoforms are expressed in mouse circumvallate taste receptor cells. Negative controls consisting of samples without reverse transcriptase lacked any visible bands, indicating that the PCR products in these experiments were not due to genomic DNA amplification. Moreover, all mRNA samples were treated with DNase prior to reverse transcription and tested for genomic contamination with a primer set for the housekeeping gene GAPDH. In addition, all the PCR products from the circumvallate taste samples in a given figure were amplified from a single cDNA sample. Therefore, if the samples contained genomic contamination, all of the PCR reactions would be expected to generate amplicons.

In some of the immunocytochemistry studies, we used a transgenic mouse which has GFP linked to the IP_3_R3 promoter. TRCs that express IP_3_R3 can be identified by their fluorescence which allows us to recognize Type II taste cells that detect bitter, sweet and umami tastants but lack conventional chemical synapses [44], [57]. While IP_3_R3 is primarily expressed in Type II cells, reports indicate that IP_3_R3 expression is not absolutely restricted to Type II cells [24], [57]. Our characterization of these transgenic mice did not find any overlap with the IP_3_R3-GFP expression and synaptic markers [44]. However, since its expression in other cell types has not been rigorously characterized and electron microscopy studies have demonstrated that almost all IP_3_R3 expressing taste cells are Type II cells [57], for this study we are presuming that the presence of IP_3_R3 identifies the taste cell as a Type II cell. Experiments were also performed in the GAD67-GFP mouse which expressed GFP fluorescence in some Type III taste cells. These taste cells have been shown to express synaptic markers and presumably have conventional synapses [24], [25].

### Expression of GABA_A_ subunits

The expression of GABA_A_ receptor subunits in mouse taste cells and non-gustatory lingual epithelium was examined using RT-PCR and immunocytochemical analysis. GABA_A_ shares a high degree of basic structural similarity and functional characteristics with other members belonging to the superfamily of ligand-gated ion channels [58]. We concentrated on the GABA_A_ α and β subunits because these subunits contribute to the ligand binding pocket and form the functional pore of the channel [59], [60], [61], [62].

**Figure 1:**
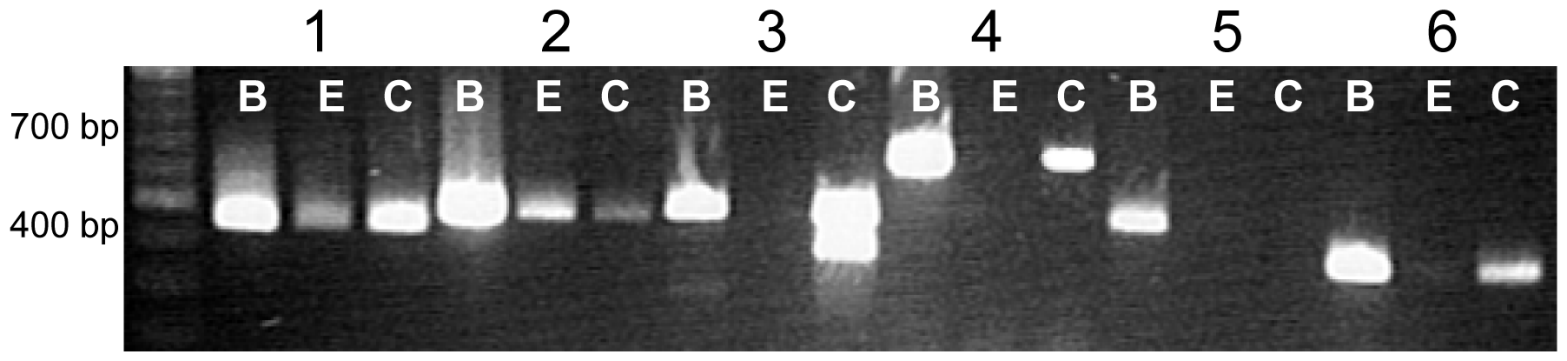
RT-PCR analysis of the GABA_A_α subunits. cDNA from circumvallate TRCs (C), non-gustatory lingual epithelium (E), and brain tissue (B) were subjected to PCR analysis using gene specific primers for the GABA_A_α subunits 1–6. PCR products were separated by agarose gel electrophoresis. Bands were observed for all subunits in the control brain tissue at the appropriate sizes (α1–455 bp, α2–498 bp, α3–511 bp, α4–699 bp, α5–465 bp, α6-358 bp) while only GABA_A_α1, GABA_A_α2, GABA_A_α3, GABA_A_α4, and GABA_A_α6 subunits were detected in taste buds. GABA_A_α1 and GABA_A_α2 were detected in the non-gustatory lingual epithelium. Results were repeated at least three times and example data are shown.

Of the six known genes that code for the GABA α isoforms, we identified five alpha (α) members expressed in the TRCs of mouse circumvallate papillae (Figure 1). In brain samples, gene specific primers for each of the GABA_A_α_1–6_ subunits amplified PCR products with the expected molecular weights (1–455bp, 2–498 bp, 3–511 bp, 4–699 bp, 5–465 bp, and 6–358 bp). In circumvallate taste buds, we detected GABA_A_α_1_, GABA_A_α_2_, GABA_A_α_3,_ GABA_A_α_4,_ GABA_A_α_6_ but not GABA_A_α_5_. Experiments were repeated at least three times to confirm these results. These data parallel the findings reported by Cao et al. [12] in the rat for GABA_A_α_1_, but they did not report any GABA_ A_α_3_ expression. This earlier study did not analyze for the potential expression of all 6 subunits, so there may be more differences in the expression of the other GABA_A_α subunits that have not yet been determined. We also detected GABA_A_α_1_ and GABA_A_α_2_ in the non-gustatory lingual epithelium but the remaining GABA_A_α isoforms were not amplified. In some samples, a truncated GABA_A_α_3_ PCR product was also amplified (see Figure 1).

**Figure 2:**
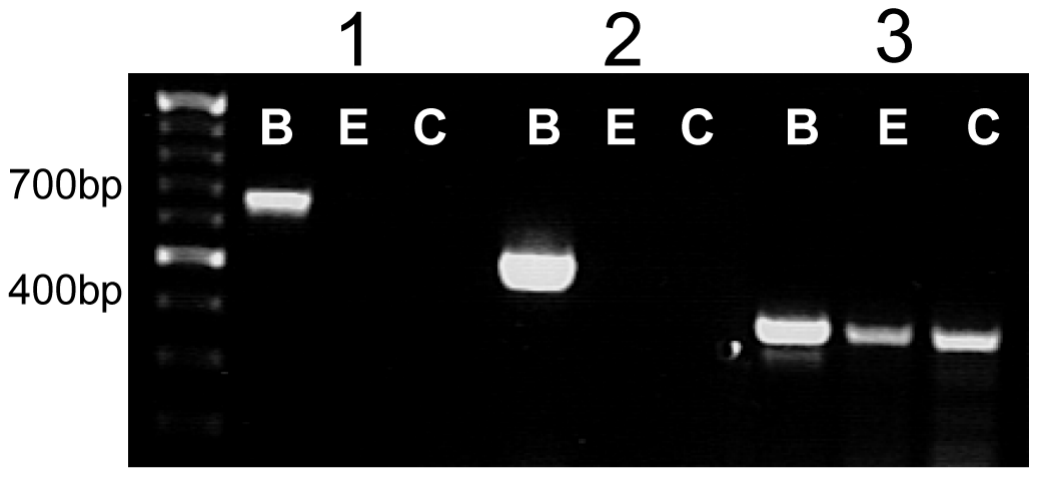
RT-PCR analysis of the GABA_A_β subunits. cDNA from circumvallate TRCs (C), non-gustatory lingual epithelium (E), and brain tissue (B) were subjected to PCR analysis using gene specific primers for the GABA_A_ ****β subunits 1–3. PCR products were separated by agarose gel electrophoresis. Bands were observed for all subunits in the control brain tissue at the appropriate sizes (β1–665 bp, β2–514 bp, β3–415 bp), but only GABA_A_β3 was amplified in the TRCs and non-gustatory epithelium. Results were repeated at least three times and representative data are shown.

We also identified the GABA_A_β subunits expressed in mouse taste cells (Figure 2). We were unable to amplify any PCR products for β1 or β2 but detected transcript for the β3 isoform from circumvallate mouse TRCs and non-gustatory epithelium. PCR products of the appropriate size were amplified the brain control sample for β1 (665 bp), β2 (514 bp) and β3 (415 bp).

**Figure 3:**
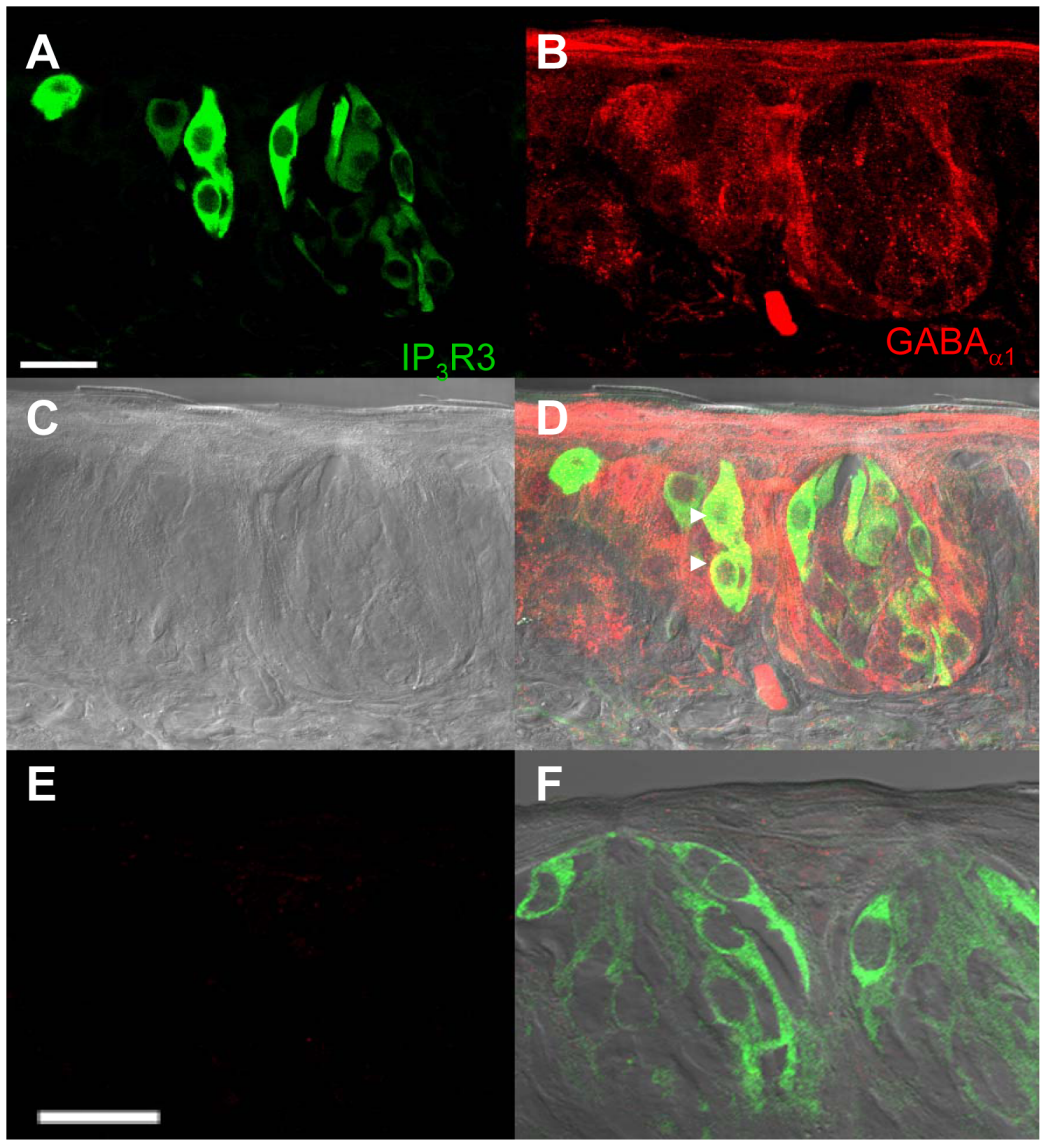
Localization of GABA_A_α1 receptors in the IP_3_R3-GFP expressing circumvallate taste buds. A Z-stack of 4 laser scanning confocal micrographs (LSCM, 0.5 µm each, collected 1 µm apart) of circumvallate taste buds from an IP_3_R3-GFP mouse labeled with an antibody directed against the GABA_A_alpha1 subunit is shown. Panel **A** shows the GFP fluorescence with the corresponding anti-GABA_A_α1 immunoreactivity of the same section shown in panel **B** (red labeling). A DIC bright field image of the taste buds is shown in **C**. An overlay of the images from **A**, **B**, and **C** is shown in **D** and demonstrates that some IP_3_R3-GFP expressing taste cells were immunoreactive for GABA_A_α1 (see arrowheads for example cells). The lack of labeling when the section is incubated with primary antibody that has been pre-incubated with blocking peptide is shown in **E.** All staining is eliminated when the blocking peptide is present. **F** shows an overlay of the panel from **E** with the corresponding DIC image and GFP expression in the taste buds. Scale bars  = 20 µm.

**Figure 4:**
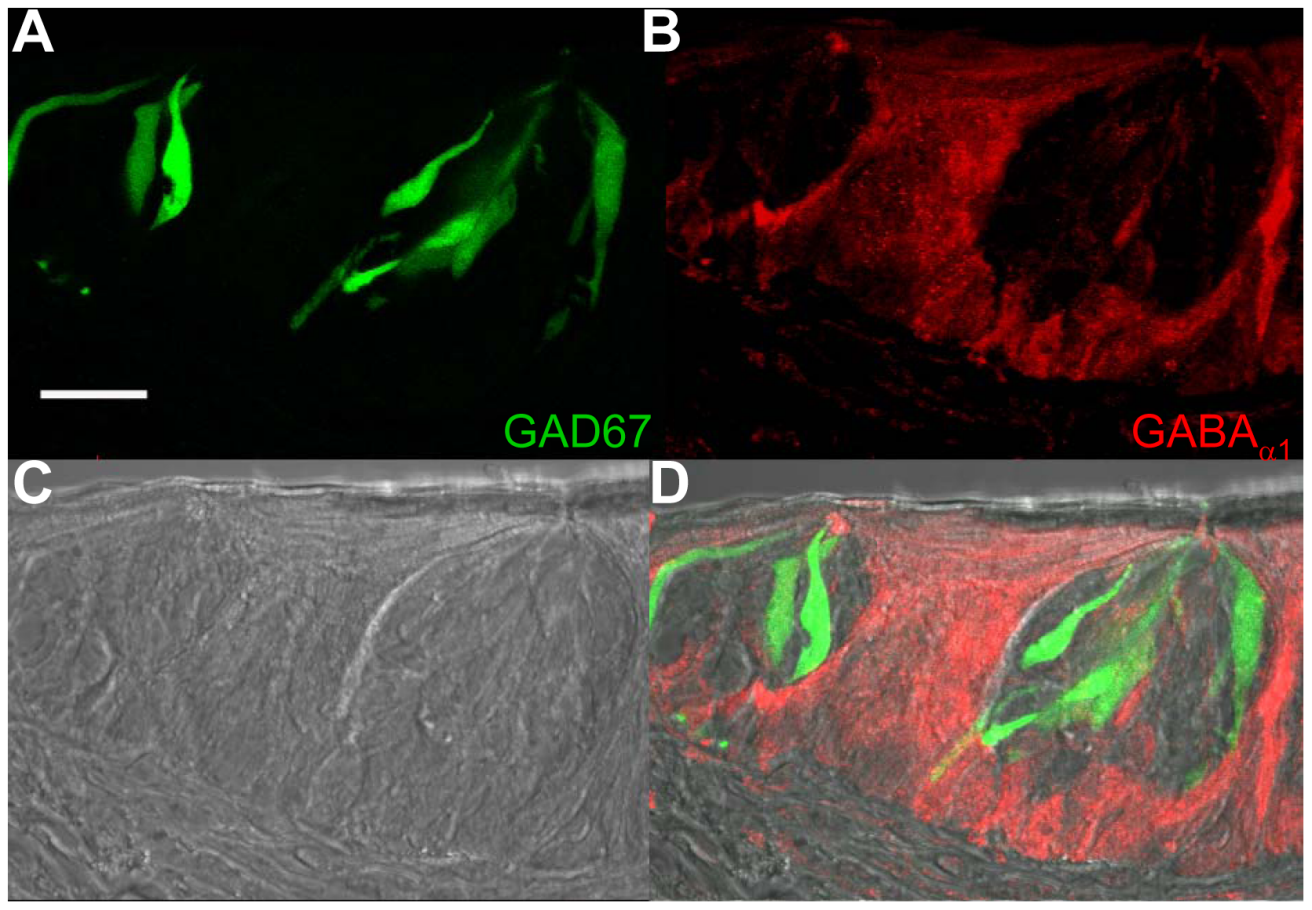
Localization of GABA_A_α1 receptors in the GAD67-GFP expressing circumvallate taste buds. A Z-stack of 5 LSCMs (0.5 µm each, collected 1 µm apart) of circumvallate taste buds from a GAD67-GFP mouse labeled with an antibody directed against the GABA_A_α1 subunit is shown. Panel **A** shows the GFP fluorescence with the corresponding anti-GABA_A_α1 immunoreactivity of the same section shown in panel **B** (red labeling). A DIC bright field image of the taste buds is shown in **C**. An overlay of the images from **A**, **B**, and **C** is shown in **D**. Negative controls are the same as shown in Figure 3. Scale bars  = 20 µm.

Immunocytochemical studies provide further evidence for the presence of the ionotropic GABA receptors in mouse circumvallate papillae. Staining of TRCs with the GABA_ A_α_1_ antibody showed a spotty distribution of GABA immunoreactivity throughout the taste cell in a subpopulation of TRCs (Figure 3A–D) with no corresponding labeling in the negative control sample (Figure 3E–F). GABA_A_ immunoreactivity was detected in all taste buds that were labeled with anti-GABA_A_ antibodies (n = 173 taste buds from IP_3_R3-GFP mice and n = 64 from GAD-GFP mice). Some GABA_ A_α_1_-immunoreactivity was detected on the IP_3_R3-GFP taste cells indicating that these receptors are expressed on some Type II taste cells. There was also GABA_ A_α_1_ labeling in the cells surrounding the taste bud. This labeling was abolished when the blocking peptide was incubated with the primary antibody (see Figure 3E) and immunoreactivity was not readily apparent below the taste bud (see Figure 3B), so we concluded the antibody labeling is specific and that GABA_ A_α_1_ expression is not restricted to the taste bud. This agrees with our RT-PCR results that found GABA_ A_α_1_ expression in both the taste buds and non-gustatory lingual epithelium. We also evaluated GABA_ A_α_1_ expression in Type III cells using the GAD67-GFP to identify this sub-population of taste cells (Figure 4). Some labeling in the taste bud was detected but the strongest labeling was in the surrounding epithelial cells. We saw a few GAD67-GFP labeled TRCs that had some immunoreactivity for GABA_ A_α_1_, but most fluorescent cells were not labeled.

### Expression of GABA_B_ subunits

**Figure 5:**
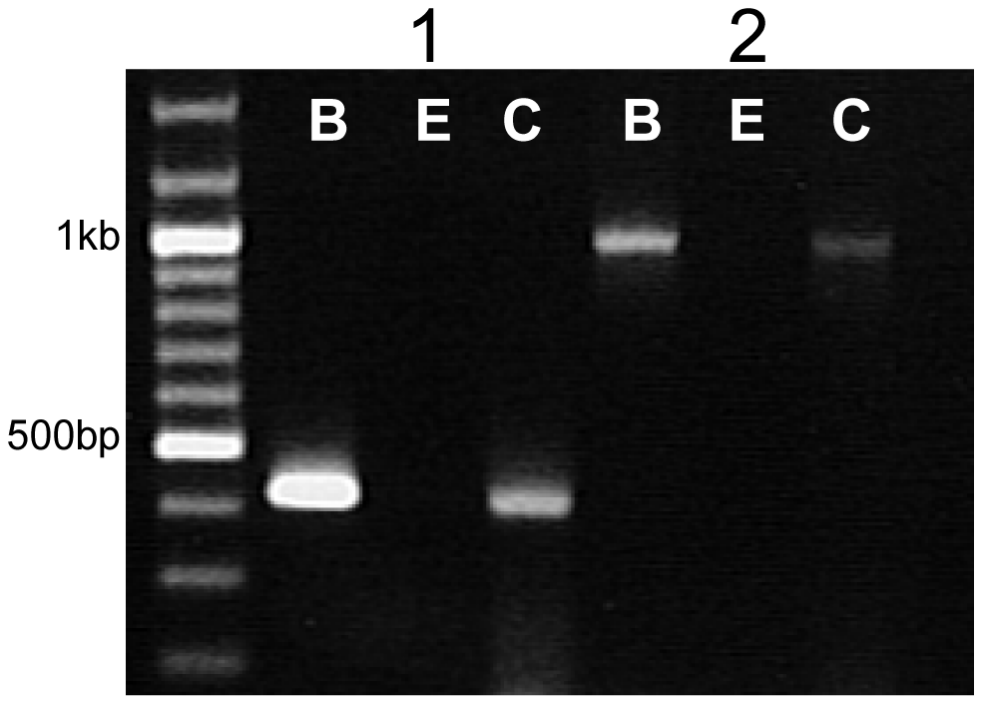
RT-PCR analysis of the GABA_B_ subunits. cDNA from circumvallate TRCs (C), non-gustatory lingual epithelium (E) and brain tissue (B) were subjected to PCR analysis using gene specific primers for the GABA_B_ subunits 1 and 2. Both subunits (B1-427 bp, B2-993 bp) were amplified in the TRCs and brain tissue but not in the lingual epithelium. Results were repeated at least three times and representative data are shown.

We also determined if circumvallate papillae TRCs express metabotropic GABA_B_ receptors. Native G-protein-coupled GABA_B_ receptors are heterodimers composed of two subunits, GABA_B1_ and GABA_B2_ [63], [64], [65]. In this heterodimer, the ligand binding domain is found only within the GABA_B1_ subunit while G protein activation is entirely mediated through the GABA_B2_ subunit. Physiological GABA_B_ receptor responses are inhibited if either subunit is nonfunctional [66], [67]. We measured for the expression of both subunits using RT-PCR and immunocytochemistry. PCR products for GABA_B1_ and GABA_B2_ in the brain control samples were the expected sizes of 427 bp and 923 bp with no amplification in the non-gustatory lingual epithelium. Similar results were obtained with cDNA isolated from mouse circumvallate taste cells, indicating the presence of a GABA_B_ heterodimer in peripheral taste cells (see Figure 5).

**Figure 6:**
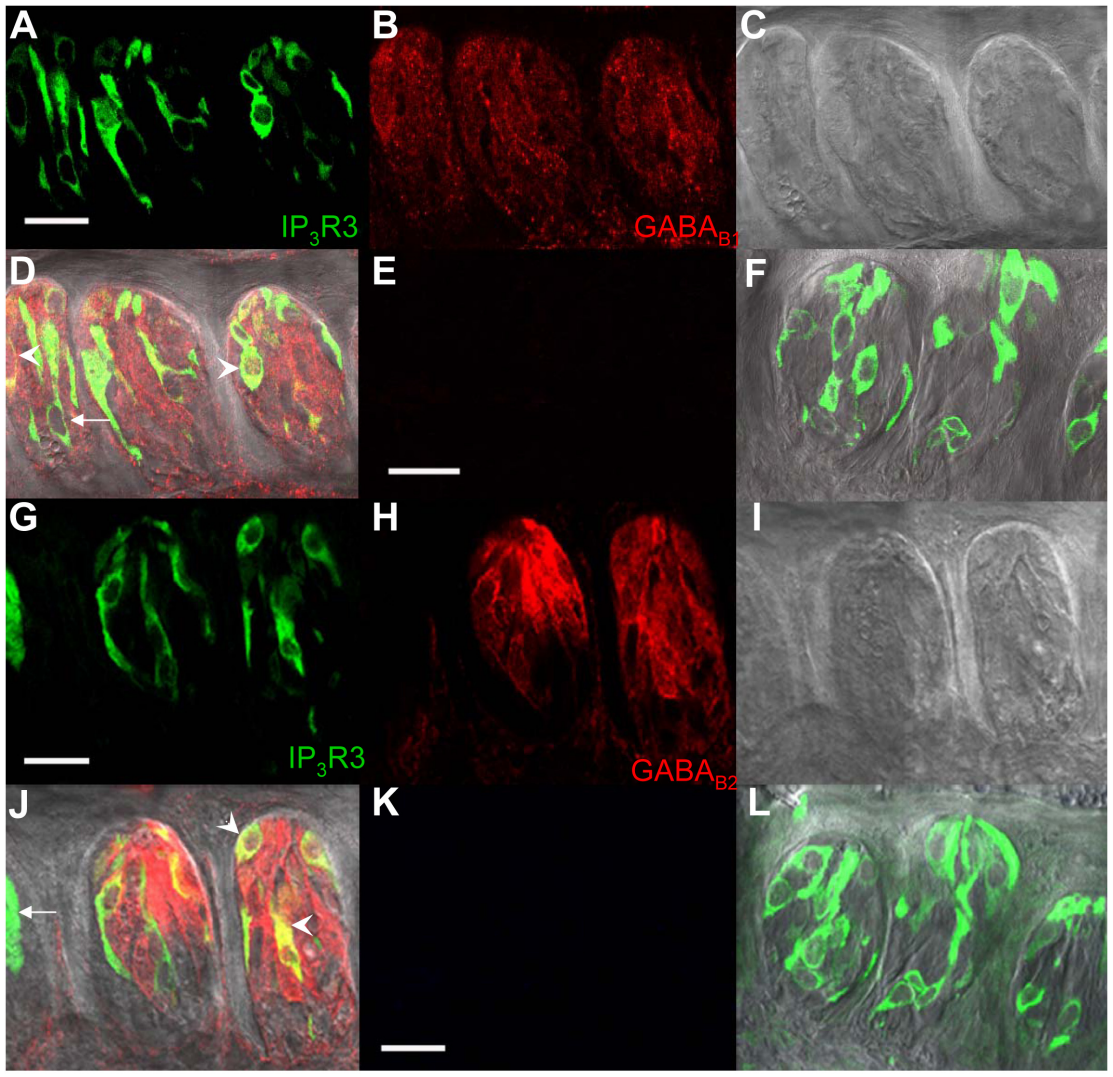
Localization of GABA_B_ receptors in the circumvallate taste buds from an IP_3_R3-GFP expressing mouse. A Z-stack of 4 LSCMs (0.5 µm each, collected 1 µm apart) of mouse circumvallate taste buds with GFP expression in the IP_3_R3 expressing taste cells is shown in panel **A**. The corresponding labeling with anti-GABA_B1_ is shown in panel **B** and the DIC image shown in **C**. **D**, When images were combined, labeling of taste cells with anti-GABA_ B1_ had some co-expression with IP_3_R3-GFP expressing taste cells (see arrowheads). Not all IP_3_R3-GFP expressing taste cells were immunoreactive (see arrow). The lack of secondary labeling in the negative control is shown in **E**. **F**, An overlay of **E** on the DIC image is shown with the corresponding GFP expression. The results from a parallel experiment using anti-GABA_ B2_ are shown in **G–J** with the appropriate negative controls in **K** and **L**. Scale bars  = 20 µm.

**Figure 7:**
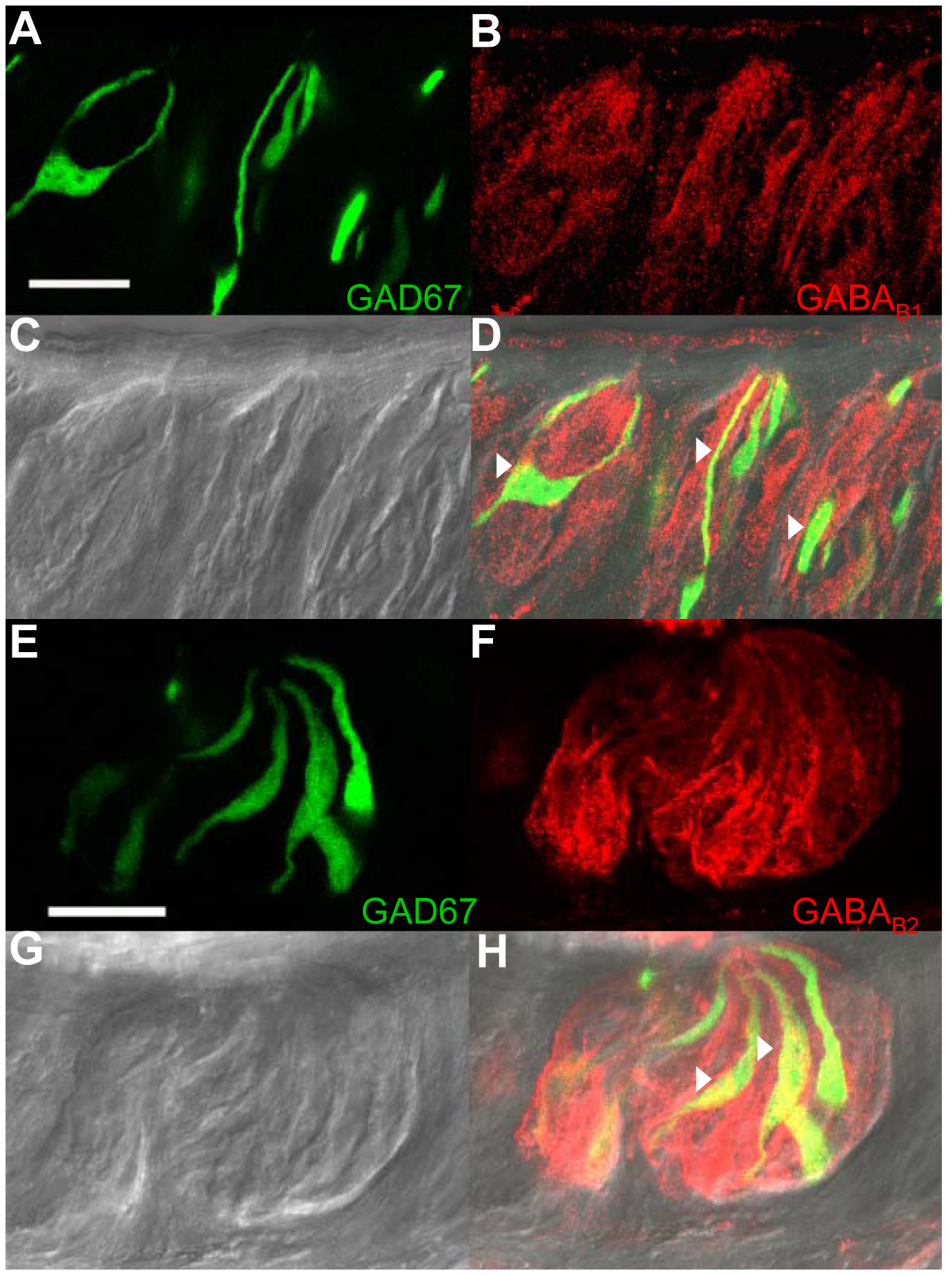
Localization of GABA_B_ receptors in the circumvallate taste buds from a GAD67-GFP mouse. A Z-stack of 4 LSCMs (0.5 µm each, collected 1 µm apart) from mouse circumvallate taste buds with GFP expression in the GAD67-expressing taste cells is shown in panel **A**. The corresponding labeling with anti-GABA_B1_ is shown in panel **B** and the DIC image shown in **C**. **D**, When images were combined, many GAD67-GFP expressing TRCs were labeled with anti-GABA_ B1_ (see arrowheads for example cells). Parallel results using anti-GABA_ B2_ are shown in **E–H**. Negative controls are the same as those shown in Figure 6. Scale bars  = 20 µm.

Immunocytochemical analysis of the GABA_B1_ expression in TRCs found antibody labeling was expressed in all the circumvallate taste buds that were analyzed (n = 77 taste buds from IP_3_R3-GFP mice and n = 43 taste buds for GAD67-GFP mice). Some immunoreactivity overlapped with the IP_3_R3-GFP expressing taste cells (Figure 6A–D, see arrowheads) while other IP_3_R3 expressing cells (see arrow) were not immunoreactive for GABA_B1_. Almost all labeling was restricted to the taste buds with very little to no labeling found in the surrounding epithelium. Negative controls (Figure 6E–F) lacked any non-specific labeling. Similar labeling patterns were detected with anti-GABA_B2_ [53] in all of the taste buds tested (Figure 6G–L, n = 109 taste buds from IP_3_R3-GFP mice and n = 72 taste buds from GAD67-GFP mice). Parallel experiments performed on circumvallate taste buds from the GAD67-GFP mouse revealed heavy co-localization of the GABA_B_ receptors and the GFP fluorescence for both B1 and B2 isoforms (Figure 7). Control immunocytochemical experiments determined that anti-GABA_B1_ and anti-GABA_B2_ were co-expressed in the same TRCs in the circumvallate papillae (See Figure S2).

### Expression of GAT transporters

**Figure 8:**
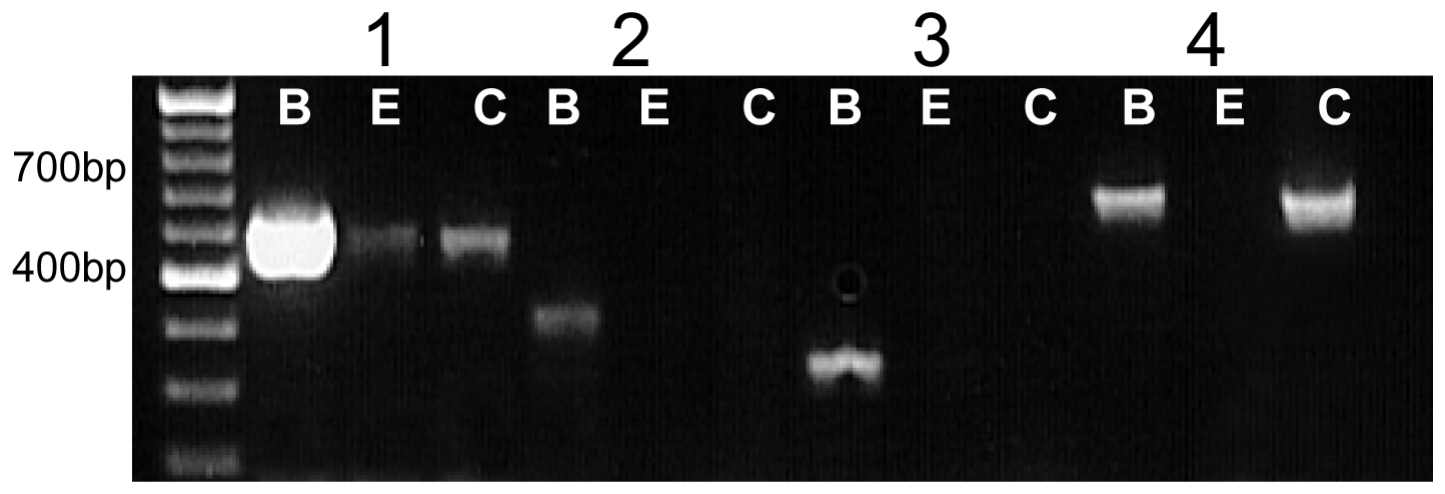
RT-PCR analysis of the GABA transporters. cDNA from circumvallate TRCs (C), non-gustatory lingual epithelium (E), and brain tissue (B) were subjected to PCR analysis using gene specific primers for the GAT transporters 1–4. PCR products were amplified for all subunits in the control brain tissue at the appropriate sizes (1–697 bp, 2–438 bp, 3–354 bp, 4–681 bp) while only GAT1 and GAT4 were detected in the taste cells. GAT1 was also detected in the non-gustatory lingual epithelium. Results were repeated at least three times and example data are shown.

In light of these data revealing the molecular machinery for both types of GABA receptors, we reasoned that GATs should be expressed in or near mouse taste buds if GABA receptors in taste buds are involved in GABAergic transmission. RT-PCR analysis (see Figure 8) revealed the presence of GAT1 (697 bp) and GAT4 (681 bp) in mouse circumvallate taste cells, but did not detect GAT2 (438 bp) and GAT3 (354 bp). GAT1 was amplified in the non-gustatory lingual epithelium sample while the other transporters were not (Figure 8).

In mice, there are four different GATs: slc6a1 (GAT1), slc6a12 (GAT2), slc6a13 (GAT3), and slc6a11 (GAT4) while in rats there are only three different proteins: SLC6A1 (GAT1), SLC6A13 (GAT2) and SLC6A11 (GAT3). The GAT1 sequences are the same in both species while the rat GAT2 is orthologous to mouse GAT3 and the rat GAT3 is orthologous to mouse GAT4 [40]. Preliminary immunocytochemical analyses with anti-GAT1 in the circumvallate taste buds were inconsistent. In one experiment, anti-GAT1 labeling was widespread throughout the taste bud and the surrounding non-gustatory lingual epithelium. This corresponds with the amplification of GAT1 mRNA from the non-lingual gustatory epithelium as well as the circumvallate taste buds. However, we were unable to repeat our results and did not include them in this study.

**Figure 9:**
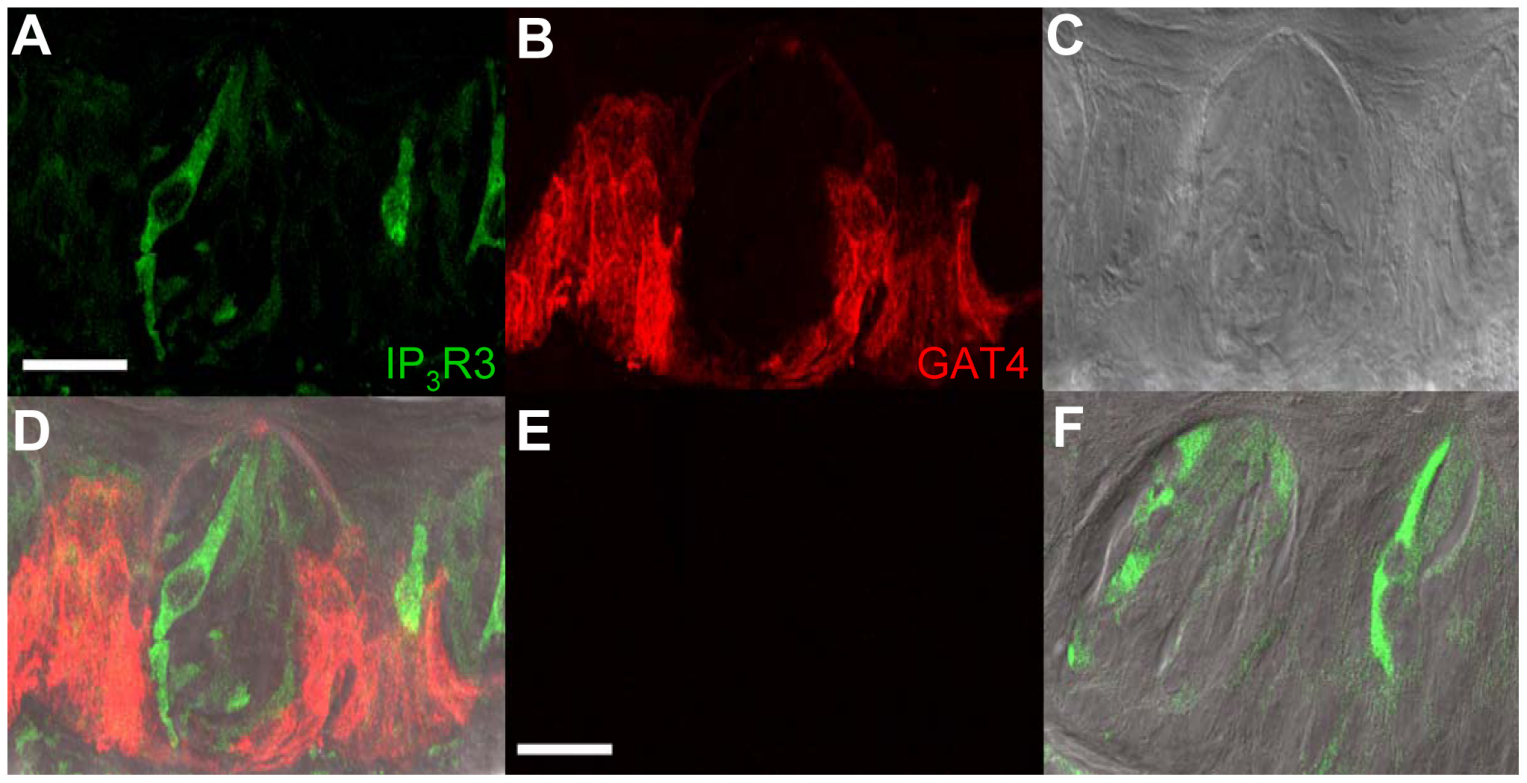
Mouse GAT4 immunoreactivity in mouse IP_3_R3-GFP expressing circumvallate papillae. A Z-stack of 5 LSCMs (0.5 µm each, collected 1 µm apart) of mouse circumvallate taste buds with GFP expression identifying the IP_3_R3-expressing taste cells is shown in panel **A**. The corresponding image of the taste buds labeled with an anti-rat GAT3 (mouse GAT-4) antibody is shown in **B** with the corresponding DIC image shown in **C**. **D**, An overlay of **A**, **B**, and **C** illustrates that most labeling is localized in the surrounding cells near the basolateral portion of the taste bud and in a few cells in basolateral portion of the taste bud. The lack of labeling in the negative control is shown in **E**. An overlay of **E**, the corresponding GFP expression and DIC image is shown in **F**. Scale bars  = 20 µm.

**Figure 10:**
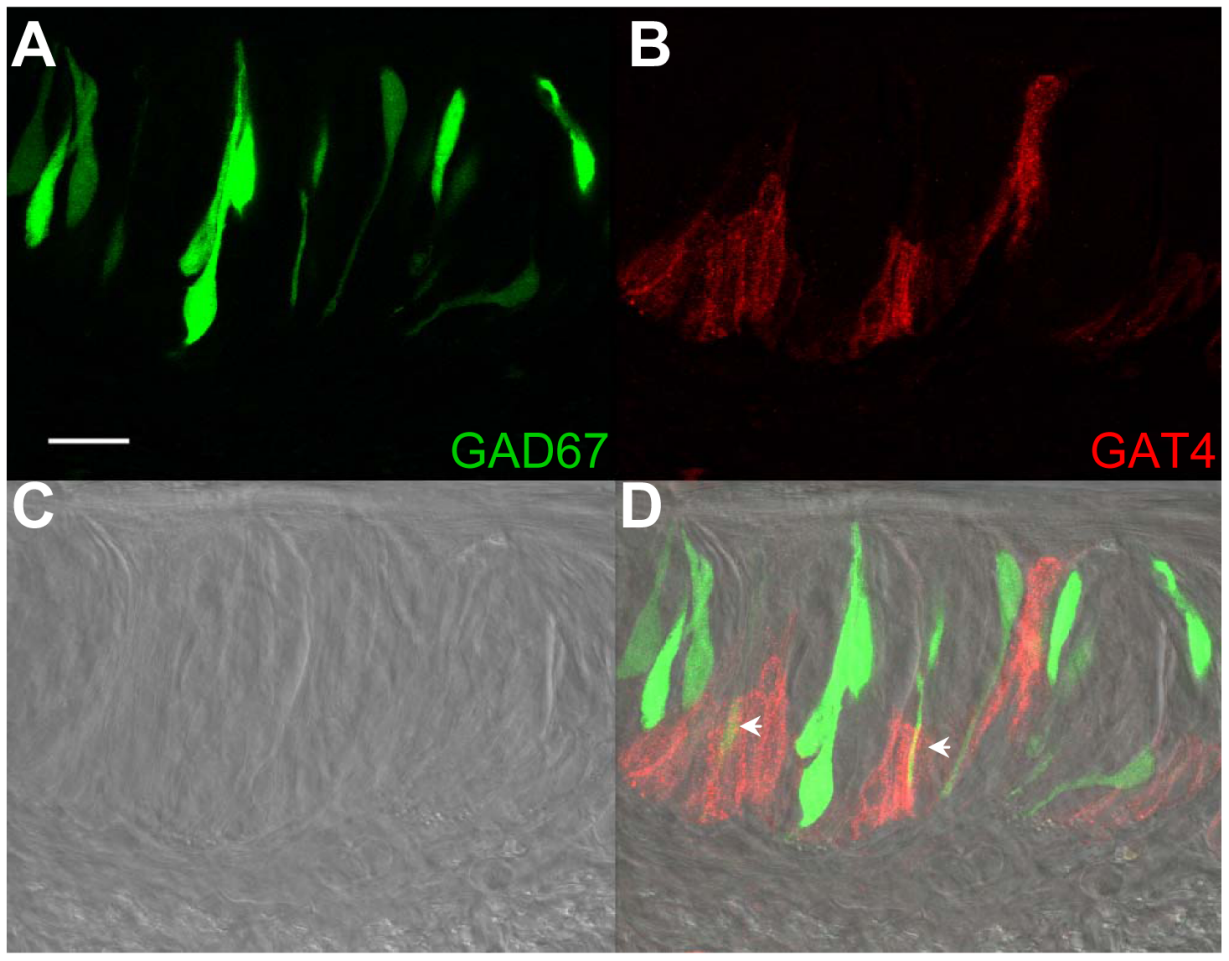
Mouse GAT4 immunoreactivity in circumvallate papillae from GAD67-GFP mice. A Z-stack of 5 LSCMs (0.5 µm each, collected 1 µm apart) of mouse circumvallate taste buds with GFP expression identifying the GAD67-expressing taste cells is shown in panel **A**. The corresponding image of the taste buds labeled with an anti-rat GAT3 (mouse GAT4) antibody is shown in **B** with the DIC image shown in **C**. **D**, The overlay of **A**, **B**, and **C** reveals that most GAT3 labeling is localized in the surrounding cells near the basolateral portion of the taste bud. A few GFP expressing taste cells have some overlap with the mouse GAT4 immunoreactivity (see arrowheads). The negative control is the same as Figure 9. Scale bars  = 20 µm.

Immunocytochemical analysis of circumvallate taste buds with anti-rat GAT3 is shown in Figure 9 and corresponds to GAT4 immunoreactivity in the mouse. The most intense anti-mouse GAT4 immunoreactivity was localized to a few TRCs in the basolateral portion of the taste bud and in the cells surrounding the taste buds. Most anti-mouse GAT4 immunoreactivity was absent within the taste bud and we did not detect any overlap between anti-mouse GAT4 labeling and IP_3_R3-GFP expressing TRCs. Similar expression patterns for anti-mouse GAT4 were found in the GAD67-GFP expressing taste cells (Figure 10). There was some overlap between the GAD67-GFP fluorescence and the anti-mouse GAT4 labeling (see arrowheads), but this was very occasional as most anti-mouse GAT4 labeling was localized in the cells surrounding the taste buds. Due to these differences in terminology between rats and mice, our GAT4 findings parallel the report of GAT3 in rat taste cells [43], including the localization of the protein at the basolateral portion of the taste buds.

## Discussion

This study is the first to systematically analyze the compositional expression of GABA receptors and transporters in mouse TRCs. Our results indicate that circumvallate taste buds express mRNA transcripts for some of the GABA_A_ ionotropic subunits (á1, á2, á3, á4, á6 and â3), both metabotropic subtypes (B1 and B2), and two GABA transporters (GAT1 and GAT4). Since functional GABA_A_ receptors must express both á and â subunits [63], [64] and GABA_B_ receptors require both B1 and B2 isoforms to function [66], [67], this study confirms that the mouse peripheral taste system has all the necessary components to utilize multiple GABAergic signaling mechanisms. GABA activity can also be mediated by the ionotropic GABA_C_ receptor, a third type of pharmacologically distinct GABA receptor. So far, studies have primarily linked the expression of GABA_C_ receptors to regions of the visual system [68], [69] and the hippocampus [70] . Nonetheless, we ran experiments to see if GABA_C_ receptor expression was apparent in TRCs but RT-PCR analysis of mRNA revealed no transcripts for this particular receptor isoform (data not shown).

We followed up our RT-PCR analysis with immunocytochemistry to determine if the GABA receptor and transporter proteins were also expressed in the peripheral taste system. These data would further support the hypothesis that GABA is an important neurotransmitter in the taste bud. Immunocytochemical analysis revealed the protein expression patterns for ionotropic and metabotropic GABA receptors as well as a GABA transporter. Taken with the report that GAD67 is expressed in a subset of mouse TRCS [24], these data suggest that GABA likely contributes to the formation or modulation of output signals from the mouse taste bud. Recent evidence has also determined that GABA as well as known modulators of GABA activity, significantly affect the physiological properties of rat taste cells [12] which provides further support for the hypothesis that GABA is a physiologically relevant neurotransmitter in the mammalian taste system.

Our immunostaining for GABA_A_α_1_ differs from results in the rat, which found GABA_A_α_1_ immunoreactivity was restricted to a small subset of TRCs and was not expressed in the surrounding epithelium [12]. Our experiments revealed labeling in the cells surrounding the taste buds as well as the labeling in the taste cells. These results were confirmed with RT-PCR analysis which identified mRNA for GABA_A_α_1_ in both taste buds and non-gustatory lingual epithelium. Use of the blocking peptide for anti-GABA_A_α_1_ eliminated this staining (Figure 3E-F) which confirms that this staining pattern is specific for GABA_A_α_1_. Since we used the same antibody that was used in the Cao et al. [12] study, these differences may be due to species differences or differences in fixation. Cao et al. [12] reported using immersion fixation in either 4% paraformaldehyde or Bouin's fixative with subsequent tissue embedding in paraffin for sectioning. We transcardially perfused the mice with 4% paraformaldehyde/0.1 M PB and embedded the tissue in OCT. We confirmed our findings using an antibody that recognizes all six GABA_A_ receptor subunits (data not shown) and obtained similar results for both antibodies.

This labeling pattern for GABA_A_α_1_ in the surrounding epithelia suggests that GABA may have additional functions outside the peripheral taste system. Earlier studies have reported the presence of GABAergic receptors, specifically GABA_A_ receptors, in multiple lung epithelial cell types [71], [72], [73]. Within this system, these GABA_A_ receptors play an essential role in regulating mucus production [72]. Their expression in the epithelia cells surrounding the taste buds suggests that these receptors may have similar roles in the tongue. GABA_A_ receptors have also been shown to have trophic roles in neuronal maturation [74] and it is possible they are exerting similar effects in the maturation of the peripheral taste receptor cells. Further studies are needed to address these questions.

Specific GABA_ A_α_1_ immunoreactivity was detected along the plasma membrane of some IP_3_R3-GFP expressing Type II taste cells which suggests that these cells are sensitive to GABA release. Since GAD67 expression has been localized to Type III taste cells in mice [24] and both Type III and gustducin expressing taste cells in rat [12], the presence of the GABA_A_ receptor on some Type II cells make them a potential target for modulation by other taste cell populations in the taste bud. This supports the hypothesis that paracrine signaling within the taste bud influences the final output signal that is sent to the brain [2], [19].

GABA_B_ receptors are expressed within the mouse taste bud with no discernable expression in the surrounding cells. Only a subpopulation of taste cells exhibited antibody labeling and negative controls were blank, indicating that the antibody labeling was specific. In addition, multiple studies in other cell types have validated that these antibodies are specific for GABA_B1_ and GABA_B2_ [51], [52], [53]. Some overlap with IP_3_R3-GFP expressing TRCs was detected but most anti-GABA_B1/B2_ labeling was present in taste cells that do not express IP_3_R3 and are not Type II cells. Since GABA_B_ receptors inhibit the activity of their target cells on a relatively slow time scale [75], this labeling pattern suggests that stimulus-evoked GABA release likely causes a longer term inhibition of a specific subset of target cells, including some Type II cells. In the rat, Cao et al [12] reported no overlap with the anti-GABA_B1_ and anti-gustducin immunoreactivity which is another Type II cell marker [76], [77]. Since gustducin is found in a subset of PLCβ2/IP_3_R3 expressing taste cells [78], we predict that the GABA_B2_ labeling is likely present in the Type II taste cells that do not express gustducin. We also determined that most, but not all, GAD67-GFP expressing Type III taste cells were labeled with the anti-GABA_B_ antibodies, indicating that GABA can function in an autocrine manner for this sub-population of Type III cells. Growing evidence suggests that the final stimulus-evoked output signal from taste cells can be influenced in a paracrine manner [2], [12], [19] and our data support a role for GABA_B_ in these processes.

Using RT-PCR analysis, we also detected GAT1 and GAT4 expression in mouse circumvallate taste cells (Figure 8). Immunohistochemical analysis using an antibody raised against GAT-1 was inconsistent and was not included. However, our mouse GAT4 immunolabeling was very consistent and was primarily restricted to the basolateral portion of a few taste cells and the cells immediately next to the taste buds with very little to no labeling in the apical portion of the taste bud. Therefore, mouse GAT4 transporters are primarily localized at the site of most synaptic activity and where one would predict most GABA signaling would occur. It has been reported that GAT2 and GAT3 are expressed in the rat taste system, with GAT3 expressed primarily at the margin of the taste bud and in some TRCs, while GAT2 had more widespread expression [43]. In mice, our RT-PCR analysis did not identify GAT2 expression but did identify the presence of GAT1 which was not reported in rat [43]. It is likely that species specific gene expression accounts for these differences. Since rat GAT3 is orthologous to mouse GAT4, our finding of mouse GAT4 expression parallels the expression pattern previously reported for GAT3 in the rat [43]. Thus, across mammalian species, these GAT transporters are localized primarily in the cells lining the basolateral portion of the taste bud, presumably where they function to remove GABA that has been released from a synapse.

The GAT1 expression in non-gustatory lingual epithelium was found using RT-PCR analysis while GAT4 was not detected. This is likely due to our method of sample collection. We isolated total RNA from epithelial tissue that had been separated from the underlying muscle and was located in an area anterior to the circumvallate papillae where no taste buds are present. While our RT-PCR data indicates that GAT1 and GABAα subunits are expressed in these epithelial cells, mouse GAT4 was not detected. Based on these data, we conclude that GAT4 is not widely expressed throughout the lingual epithelium but is preferentially associated with the taste buds. Therefore, GAT4 was not detectable in our non-lingual gustatory epithelium sample because there were no taste buds near the area where our epithelial sample was collected. While we removed the taste buds from the surrounding epithelium to isolate taste bud RNA for the RT-PCR analysis, it is possible that some surrounding epithelial cells were also collected. Since we detected very few GAT4 immunoreactive taste cells with the anti-mouse GAT4 antibody but were able to detect GAT4 using RT-PCR, it is possible that our taste bud sample was contaminated with a few of the cells that surround the taste buds.

The evidence for the role of GABA as a neurotransmitter in the taste system is accumulating. A study of GAD67-GFP knock-in mice found a strong GFP signal in taste cells which was confirmed using immunocytochemical analysis [79]. Physiological actions of GABA activity in GABA_A_ and GABA_B_ receptors have been successfully recorded in rat taste cells [12]. Our data establish the presence of both ionotropic and metabotropic GABA receptors in the mouse taste system, which may play a critical role in its responsiveness to multiple stimuli. Moreover, a GABA reuptake system which is critical to the physiological function of a GABAergic signaling pathway is expressed in the basolateral portion of the taste buds, the primary site of interaction between taste receptor cells and their post-synaptic targets. All of these data are consistent with a role for GABA as one of the neurotransmitters that regulates signaling to and from taste cells.

## Supporting Information

Figure S1 Amplification of GAPDH was used to measure for any genomic DNA contamination. All RNA from the brain and taste samples were DNAse treated and analyzed to ensure that no contaminating genomic DNA was present before being used for PCR analysis. The panel to the left illustrates the lack of GAPDH amplification in a sample that did not have any genomic DNA while the panel to the right reveals the presence of contaminating genomic DNA. When genomic DNA was detected, the sample was discarded and not included in the analysis.
(0.18 MB TIF)

Figure S2 GABA B1 and GABA B2 are co-expressed in the same taste cells. Sections from mouse circumvallate papillae were subjected to double-labeling using anti-GABA B1 (A) and anti-GABA B2 (B). C, An overlay of the images in A and B revealed similar labeling patterns for each of these antibodies.
(1.73 MB TIF)
