# Supplementary figures and images for: Expression of GABAergic Receptors in Mouse Taste Receptor Cells
Source: PLoS One. 2010 Oct 26;5(10):e13639. doi: 10.1371/journal.pone.0013639 (PMC2964312; doi:10.1371/journal.pone.0013639)

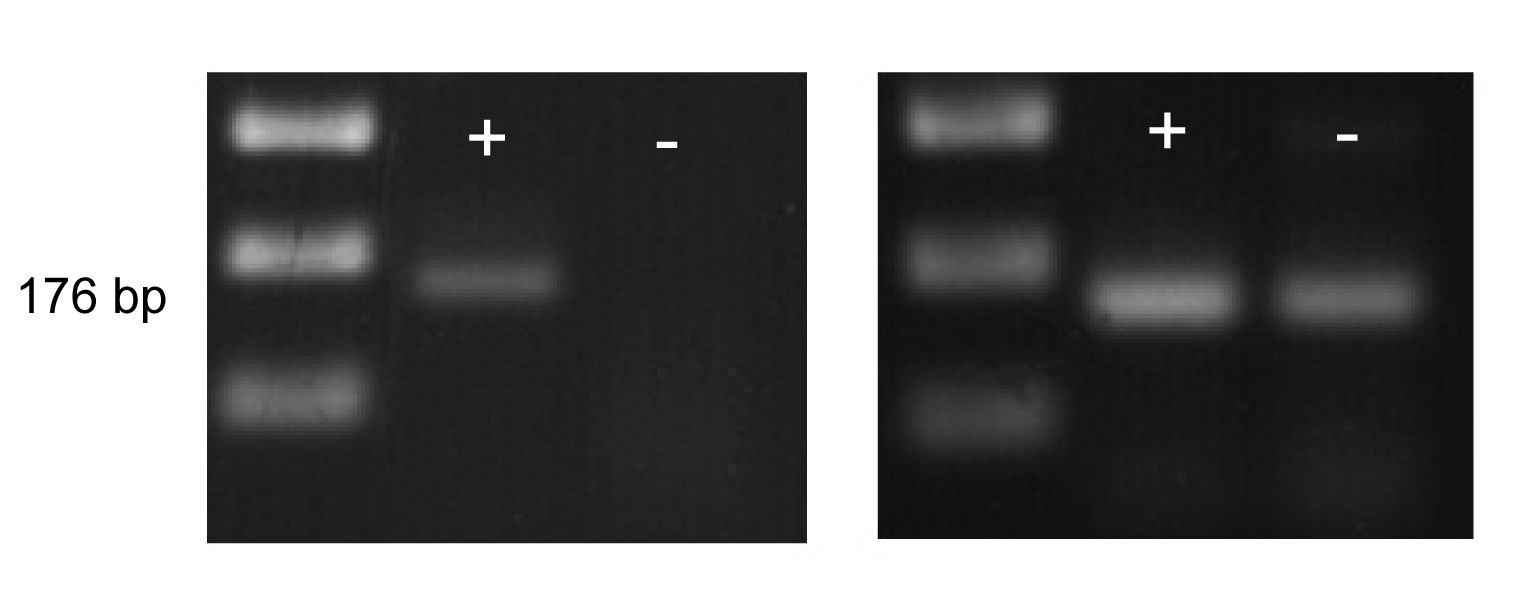

Supplement: Figure S1 — Amplification of GAPDH was used to measure for any genomic DNA contamination. All RNA from the brain and taste samples were DNAse treated and analyzed to ensure that no contaminating genomic DNA was present before being used for PCR analysis. The panel to the left illustrates the lack of GAPDH amplification in a sample that did not have any genomic DNA while the panel to the right reveals the presence of contaminating genomic DNA. When genomic DNA was detected, the sample was discarded and not included in the analysis. (0.18 MB TIF) [file pone.0013639.s001.tif]

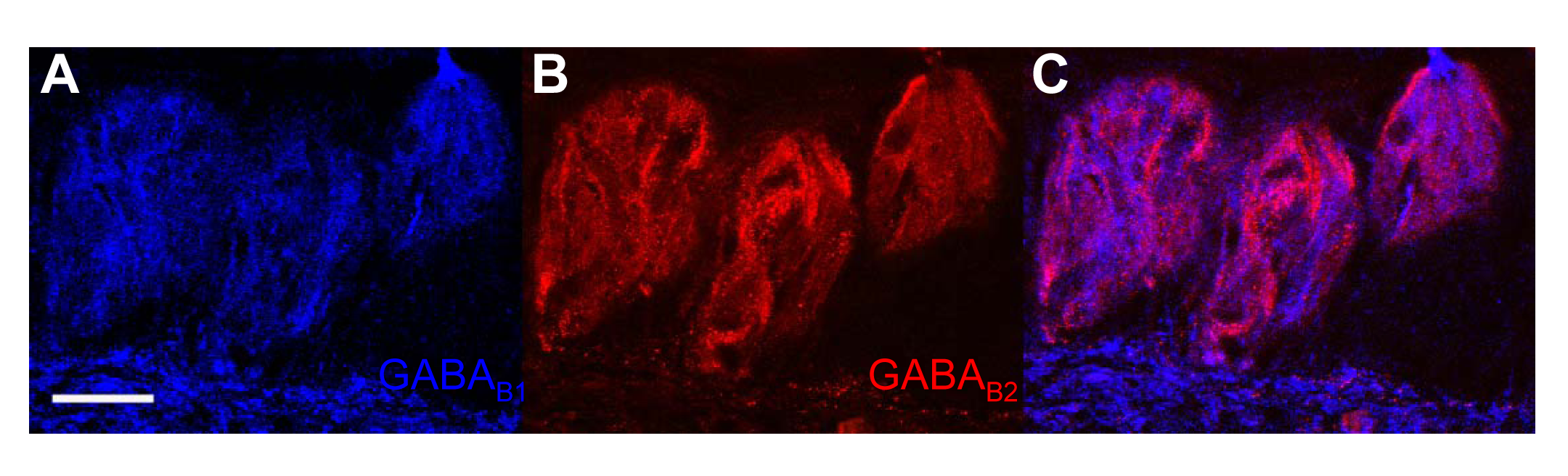

Supplement: Figure S2 — GABA B1 and GABA B2 are co-expressed in the same taste cells. Sections from mouse circumvallate papillae were subjected to double-labeling using anti-GABA B1 (A) and anti-GABA B2 (B). C, An overlay of the images in A and B revealed similar labeling patterns for each of these antibodies. (1.73 MB TIF) [file pone.0013639.s002.tif]
